# 

**Published:** 1789

**Authors:** 


### CONTENTS.

Page

I. *AN Account of an epidemic Fever that prevailed in Cornwall in the Year* 1788. *By* William May, *M. D. Extra-Licentiate of the College of Physicians, London; and Physician at Truro in Cornwall.* - 117

II. *A Case of Hepatitis; with Remarks. By Mr.* George Wilkinson, *Surgeon at Sunderland, Member of the Royal College of Surgeons, and Honorary Member of the Chirurgo-Physical Society of Edinburgh.* - - 142

III. *Farther Remarks on the Efficacy of Blue Vitriol in the Cure of Dropsy. By* William Wright, *M. D. F. R. S. and of the Royal College of Physicians and Royal Society of Edinburgh.* - - - - - 149

IV. *Some Account of the medicinal Properties of a Bark lately procured from South America. By* J. Ewer, *M. D. Physician in Trinidad.* - - - - - - - 154

V. *Farther Account of the Bark described in the preceding Article; being an Extract of a Letter from* Alexander Williams, *M. D. Physician in Trinidad.* - - - 158

Page

VI. *An Account of a Method of performing the Operation of Lithotomy at two different Times. By* Petrus Camper, *M. D. F. R. S. Honorary Professor of Physic, Anatomy, and Surgery at Amsterdam, Fellow of the Royal College of Physicians and of the Royal Society of Edinburgh, of the Imperial Academy at St. Petersburgh, and of the Royal Academy of Sciences and Royal Medical Society at Paris,* &c. *Translated from the Dutch.* - 162

VII. *An Account of a remarkable Transposition of the Viscera in the Human Body. By* Matthew Baillie, *M. B. From the Philosophical Transactions, Vol.* LXXVIII.; *with some Alterations and Additions by the Author.* 178

VIII. *An Account of the Method of making a Wine, called by the Tartars Koumiss; with Observations on its Use in Medicine. By* John Grieve, *M. D. F. R. S. Edin. and late Physician to the Russian Army. From the Transactions of the Royal Society of Edinburgh, Vol.* I. - - - - - - 197

CATALOGUE *of* BOOKS. - - 210

